# *RET* Mutational Spectrum in Hirschsprung Disease: Evaluation of 601 Chinese Patients

**DOI:** 10.1371/journal.pone.0028986

**Published:** 2011-12-09

**Authors:** Man-Ting So, Thomas Yuk-Yu Leon, Guo Cheng, Clara Sze-Man Tang, Xiao-Ping Miao, Belinda K. Cornes, Diem Ngoc Ngo, Long Cui, Elly Sau-Wai Ngan, Vincent Chai-Hang Lui, Xuan-Zhao Wu, Bin Wang, Hualong Wang, Zheng-Wei Yuan, Liu-Ming Huang, Long Li, Huimin Xia, Deli Zhu, Juncheng Liu, Thanh Liem Nguyen, Ivy Hau-Yee Chan, Patrick Ho-Yu Chung, Xue-Lai Liu, Ruizhong Zhang, Kenneth Kak-Yuen Wong, Pak-Chung Sham, Stacey S. Cherny, Paul Kwong-Hang Tam, Maria-Mercè Garcia-Barcelo

**Affiliations:** 1 Department of Surgery, University of Hong Kong, Hong Kong, China; 2 Department of Psychiatry, University of Hong Kong, Hong Kong, China; 3 Centre for Reproduction, Development and Growth, University of Hong Kong, Hong Kong, China; 4 Genome Research Centre of the University of Hong Kong, Hong Kong, China; 5 Department of Epidemiology and Biostatistics, School of Public Health, Tongji Medical College, Huazhong University of Science and Technology, Wuhan, China; 6 Department of Surgery, Guiyang Medical College Affiliated Hospital, Guiyang, China; 7 Shenzhen Children's Hospital, Shenzhen, China; 8 Changchun Children's Hospital, Changchun, China; 9 Shengjing Hospital, China Medical University, Shenyang, China; 10 Beijing BAYI Children′s Hospital, Beijing, China; 11 Capital Institute of Pediatrics, Beijing, China; 12 Guangzhou Women and Children's Medical Centre, Guangzhou, China; 13 Department of Pediatric Surgery, The First Affiliated Hospital of Sun Yat-Sen University, Guangzhou, China; 14 Department of Human Genetics, National Hospital of Pediatrics, Hanoi, Vietnam; University of Oklahoma and Oklahoma Medical Research Foundation, United States of America

## Abstract

Rare (RVs) and common variants of the *RET* gene contribute to Hirschsprung disease (HSCR; congenital aganglionosis). While *RET* common variants are strongly associated with the commonest manifestation of the disease (males; short-segment aganglionosis; sporadic), rare coding sequence (CDS) variants are more frequently found in the lesser common and more severe forms of the disease (females; long/total colonic aganglionosis; familial).

Here we present the screening for RVs in the *RET* CDS and intron/exon boundaries of 601 Chinese HSCR patients, the largest number of patients ever reported. We identified 61 different heterozygous RVs (50 novel) distributed among 100 patients (16.64%). Those include 14 silent, 29 missense, 5 nonsense, 4 frame-shifts, and one in-frame amino-acid deletion in the CDS, two splice-site deletions, 4 nucleotide substitutions and a 22-bp deletion in the intron/exon boundaries and 1 single-nucleotide substitution in the 5′ untranslated region. Exonic variants were mainly clustered in RET the extracellular domain. *RET* RVs were more frequent among patients with the most severe phenotype (24% *vs.* 15% in short-HSCR). Phasing RVs with the *RET* HSCR-associated haplotype suggests that RVs do not underlie the undisputable association of *RET* common variants with HSCR. None of the variants were found in 250 Chinese controls.

## Introduction

Hirschsprung's disease (HSCR) is a developmental disorder characterized by the absence of ganglion cells in lower digestive tract. Aganglionosis is attributed to a disorder of the enteric nervous system (ENS) whereby ganglion cells fail to innervate the lower gastrointestinal tract during embryonic development. The disease can be classified according to the length of the aganglionosis into short segment (S-HSCR; aganglionosis up to the upper sigmoid colon; 80% of HSCR cases), long segment (L-HSCR; aganglionosis up to/beyond the splenic flexure), and total colonic aganglionosis (TCA) forms. HSCR can manifest isolated or syndromic. There is significant racial variation in the incidence of the disease, and it is most often found among Asians (2.8/10,000 live births). HSCR usually presents sporadically (80% of the cases), and is most frequent in males (M∶F = 4∶1). Familial HSCR shows a non-Mendelian inheritance pattern [1].

Both rare and common germ-line *RET* variants play a role in HSCR. While *RET* common variants are strongly associated with the commonest manifestation of the disease (male, S-HSCR and sporadic forms), rare coding sequence variants (CDS-RV) are more frequently found in the less common and more severe forms of the disease (females, L/TCA-HSCR and familial) [2]. These CDS-RVs have reduced, sex-dependent penetrance and account for ∼50% of the familial and for ∼20% of the sporadic cases. Over 200 *RET* CDS-RVs (www.hgmd.cf.ac.uk) have been described in HSCR. These HSCR *RET* CDS-RVs mainly cause loss-of function of the *RET* protein, a tyrosine kinase receptor. Critically, *RET* gain-of function CDS-RVs are directly implicated in hereditary thyroid cancers [3].

Here we present the screening of the *RET* CDS-RVs in 601 Chinese HSCR patients, the largest number of patients ever reported.

## Results

In this study we report those *RET* CDS-RVs (including intron/exon boundaries) that were not found in our controls. The overall frequency is <1%. Exception is R114H, which is present in 38 patients (6.32%) but no in controls. R114H is a founder mutation within Chinese HSCR patients [4].

### *RET* variants identified in 601 Chinese HSCR patients

We identified **61 different heterozygous** RVs distributed in **100** HSCR patients (**16.64%**) (Tables 1 and S4, Tables S1(A)-S1(B)). These include 14 silent, 29 missense, 5 nonsense, 4 frame-shifts, and one in-frame amino-acid deletion in the CDS, 3 deletions and 4 nucleotide substitutions in the intron/exon boundaries and 1 nucleotide substitution in the 5′ untranslated-region (UT).

**Table 1:** Distribution of the *RET* variants across the different HSCR types.

	Short		Long		TCA		TIA	Undetermined	
HSCR patients (N = 601)	(N = 382; 63.56%^a^; [42] {15})		(N = 48; 7.99%^a^; [4])		(N = 22; 3.66%^a^; [3] {1})		(N = 1; 0.17%^a^; [1])	(N = 148; 24.63%^a^; [1])	
	Rare variants		Rare variants		Rare variants		Rare variants	Rare variants	
	Yes (9.82%^a^)	No	Yes (1.50%^a^)	No	Yes (1.16%^a^)	No	Yes (0.17%^a^)	Yes (3.99%^a^)	No
Males (N = 490; 81.53%)	49 [4] (15.46%^b^)	268 [30] {12}	6 (17.65%b) **** [**1**]	28 [2]	4 (25.00%^b^)	12 [3] {1}	0	20 (16.26%^b^)	103
Females (N = 111; 18.47%)	10 [**2**] {1} (15.38%^b^)	55 [6] {2}	3 (21.43%^b^)	11 [1]	3 (50.00%^b^)	3	1 [**1**]	4 (16.00%^b^)	21[1]
	59 [**6**] {1} (15.45%^c^)	323 [36] {14}	9 (18.75%^c^) [**1**]	39 [3]	7 (31.82%^c^)	15 [3] {1}	1 [1] (100.00%^c^)	24 (16.22%^c^)	124[1]

N: number of individuals; S: short segment aganglionosis; L: long segment aganglionosis; TCA: total colonic aganglionosis; TIA: total intestinal aganglionosis;

a : % of the total number of patients;

b : % of the total number of patients of the same sex with the same length of aganglionosis;

c : % of the total number of patients with the same length of aganglionosis; []: patients with associated anomalies; {}: patients with Down syndrome.

Five variants (R114H/R313Q/V292M/P841P/R180X) are in the dbSNP and one (T278N) in both dbSNP and 1000 Genomes Project. No population/frequency was reported (March/2011). Excluding R114H, no parental DNA was available to determine whether those “database-reported” variants identified in our study had been inherited from unaffected parents or arisen “*de novo*”.

We identified 6 patients with *de novo* events, 5 with maternally inherited and 6 with paternally inherited variants. Four patients harboured 2 CDS-RVs.

Excluding those patients included in the GWAS, whole *RET* deletion in patients homozygous for *RET* SNPs was not evaluated.

### Rare variants in other HSCR series

While 50 RVs identified in this study are novel, 6 (R180X/T278N/R313Q/E480K/Y1062C/M1064T) have been reported in other HSCR patients (Chinese/other ethnicities) and 2 (R114H/V292M) in individuals with other *RET* related disorders (Material S1).

Y1062C and M1064T have been extensively studied [5]. While Y1062C affects the RET downstream signalling no RET disruption has been demonstrated for M1064T [6]. This is in line our bioinformatics prediction (Table S2) whereby Y1062C is predicted damaging and M1064T benign. The patient reported here harbouring Y1062C (C16C_Male_L-HSCR) was born to unaffected parents and had a distant relative affected. C16C also has V397M in *cis*. We could not determine whether these variants are *de novo*, yet the fact that Y1062C has been reported advocates for an inherited mutation that segregates with reduced penetrance. The recurrence of *RET* CDS-RVs in ethnically different patients suggest that these events occurred before the European-Asian split.

Knowing whether *RET de novo* variants recur or, whether an amino-acid residue is more prone to DNA changes should be useful in the identification of putative *RET* mutation hot-spots. R180 residue (CGA) has repeatedly been altered leading to different types of mutations (R180X [7]; R180P [8] and R180Q [9]). The same is true for R114 and T278. Specific RET residues are inherently more prone to mutations than others.

### *RET* coding sequence rare variants (CDS-RVs) distribution across the different HSCR subtypes

The distribution of the *RET* CDS-RVs was analysed across different HSCR categories. HSCR patients were therefore stratified according to i) length of the aganglionic segment -severity of the HSCR phenotype-; ii) presence/absence of associated anomalies/syndrome; iii) presence/absence of familial involvement.

#### Length of the aganglionic segment

When patients were stratified according to the length of the aganglionic segment, the highest frequency of CDS-RVs was found among patients with the most severe forms of the disease (Table S1A). This is in accordance with our previous report [2]. Likewise, the highest frequency of CDS-RVs was found among females, and this became more obvious as the severity of the HSCR phenotype increased (Table 1 and Tables S1A and S1B).

We then investigated whether there was any correlation between type of *RET* mutation and severity of the HSCR phenotype: among the 17 “mutated” patients affected with the most severe phenotype, 8 (47.07%) had missense changes or in-frame deletions; 5 (29.41%) had non-sense or frame-shifts; 2 (11.76%) had silent variants and 2 (11.76%) had intronic substitutions. Among the 59 “mutated” S-HSCR patients, 48 (81.36%) had missense changes; 3 (5.08%) had non-sense or frame-shifts; 6 (10.17%) had silent changes, and 2 (3.39%) had variants in the intronic or 5′UT regions (Table S3).

#### Syndromic patients

Of the 67 syndromic patients, 8 had *RET* CDS-RVs of varying degrees of “pathogenicity”.

#### Familial cases

Three of the 4 patients with an affected distant relative harboured damaging CDS-RVs variants. This is in accordance with familial cases being enriched with severe mutations sufficient to lead HSCR [2], [10].

### Distribution of the coding sequence rare variants (CDS-RVs) across the RET gene

It is assumed that HSCR *RET* CDS-RVs are scattered all over the gene. Yet, some reports indicate that HSCR *RET* CDS-RVs tend to cluster in the extracellular domain of the protein [8], [11], [12]. “Exonic” variants (N = 55) where in excess in the sequence encoding the extracellular domain (EC) of the protein. Thirty-six (65.45%) variants were in the EC; 3 (5.45%) in the trans-membrane domain and 16 (29.09%) in the intracellular domain (Table S3).

Exon 5 (cadherin-domain) was the exon with more variants (N = 9) followed by exon 3 (N = 7). Similar data have been reported by others [8] and whether this is related to the high recombination rates reported for this exon needs further investigation [2].

The distribution of variants across *RET* is relevant as the functional consequences of missense CDS-RVs correlate with their position in the sequence [5], [13], [14]. However, after over 200 *RET* deleterious variants reported, no correlation could ever be established which reflects the contribution of other genetic factors to the disease including that of HSCR-associated *RET* common variants/SNPs.

### Assessment of the functional effect of the *RET* variants

Missense CDS-RVs were assessed for their effect in the protein. Benign missense CDS-RVs, silent and intronic variants, were scrutinized for interference with i) signature elements that govern the splicing machinery (exonic/intronic splicing enhancer/silencer sequences -ESE/ESS-), and ii) RNA stability, structure and folding (Table S2).

Out of 29 missense variants identified, 10 were predicted damaging by both Polyphen and SIFT, 16 were predicted damaging by Polyphen and tolerated by SIFT and 3 tolerated by both software packages.

Except L465L, all silent variants were predicted to hinder splicing. Similarly, all benign non-synonymous/missense variants as well as the “in-frame” deletion G731del were predicted to interfere with the splicing machinery. Four intronic variants were predicted to affect splicing by either disrupting the splice sites (acceptor/AS or Donor/DS) or the branch point (BP). The 5′UT variant did not alter any regulatory site.

As the effect of point mutations may be dependent on conservation at both DNA and protein levels, we produced conservation scores for each variation site (Table S2). Seventeen DNA sites had the maximum score implying that changes on those sites are not tolerated. At protein level, the most evolutionary conserved amino-acids are 4 residues with silent changes (*de novo*G954G/L651L/P841P/L1077L).

Silent variants with the highest conservation scores at DNA (Q327Q/L465L/L1077L) and/or amino-acid level (L651L/P841P/G954G) and non-synonymous/missense that overlapped with functional RNA structures (F961L/Y1062C) according to the UCSC Evofold track were evaluated for their impact on RNA secondary structure/stability. All but Q327Q were found to alter the wild-type RNA stem loop (Figure S1). The only silent variant with no predicted “damaging” effect thus far (L465L) was found to alter such RNA structure.

Bioinformatics prediction indicates that all variants identified but one (c.-37G>C) potentially affect the RET function in a quantitative and/or qualitative manner.

### *RET* rare variants are not necessarily on the RET HSCR-risk haplotype

A common *RET*-haplotype highly associated with HSCR comprising a functional variant (rs2435357 C/T; T = HSCR associated allele) in intron 1 is associated with reduced expression of *RET* both *in vitro* and *in vivo* [2], [15], [16], [17], [18]. The CDS-RVs *cis*/t*rans* location with respect to this low-expressing risk-haplotype is relevant to the effect and/or penetrance of the variant as the amount of mutated protein synthesized (hence the effect) would depend on the haplotype where the CDS-RVs resides. The CDS-RVs *cis/trans* location could also explain the *RET* SNPs association with HSCR by means of synthetic association [19]. Out of the **100** HSCR patients with CDS-RVs unique to HSCR (Table S4), **68** were homozygous for the *RET-*risk-haplotype (TT), **9** were homozygous for the wild-type counterpart (CC) and **23** were heterozygous (CT). Of these **23** heterozygous patients, we were able to phase their mutation with the haplotype for **15** (Material S1) [2], [17], [20]. There is not excess of risk-haplotype among patients with CDS-RVs when compared to the whole patient group (χ^2^ = 0; *p* = 0.99), indicating that the occurrence of *RET* CDS-RVs is independent of the haplotype background. *RET* CDS-RVs do not seem to contribute to the strong association of the *RET* SNPs with HSCR.

## Discussion

This is the largest *RET* sequencing study of HSCR patients ever conducted. Its aim is to provide a catalog of the *RET* coding sequence rare variants present in Chinese HSCR patients and assess the distribution of those variants across the different types of HSCR patients. In line with previous observations [2], we found that *RET* CDS-RVs are more frequently found in the less common and more severe forms of the disease (females, L/TCA-HSCR and familial). As in many other diseases, one would expect a correlation between phenotype and location of the causal variant in the gene and this was not the case. Similarly, the phenotypic variability observed among patients bearing the same variant stresses the difficulty in establishing genotype-phenotype correlations. Undoubtedly, other genetic factors affect the penetrance of *RET* rare variants and contribute to the phenotypic variability [10], [20].

We used bioinformatics tools to predict the possible implications of these variants on the gene function. Importantly, all variants (but one) were predicted to affect the protein function at different levels. The prediction of missense mutations should perhaps be the most straight forward, yet, discrepancies between Polyphen and SIFT seem to be the norm rather than the exception [21]. More daunting is to assess the predictions provided for silent and/or regulatory variants. However, bioinformatics claims cannot categorically be dismissed no fully accepted and this is widely exemplified in the literature. For instance, 19 genes were found to have truncating variants in controls in the resequencing of X-chromosome exons in mental retardation [22]. On the other hand, silent and/or regulatory RVs are often neglected and these variations underlie as many as 50 diseases [23]. Functional validation and/or faithful segregation of variants with the disease phenotype (which would only be achievable among the scarce familial cases with fully penetrant variants) are needed to fully assess the impact of those variants on the phenotype. Thus, in spite of the ambiguity regarding the potential pathogenicity of many of the variants found and based on the indisputable role of *RET* in HSCR we suspect that all RVs found are damaging. With a few exceptions, the rare *RET* RVs presented here are unique to HSCR patients and the existence of some predicted deleterious variants in the general population (1000 Genome project) only confirms that additional genetic/environmental factors are in some instances needed for the disease to occur. As the majority of causal *RET* variants described thus far are heterozygous it would be tempting to speculate that the manifestation of the disease may be dependent on factors affecting the expression of the *RET* allele in which the variant resides. We have phased the rare variants with the *RET* risk-haplotype and conclude that the undisputable association of *RET* common variants with HSCR is not due to rare variants that went undetected in our previous GWAS study. Thus, this indicates that common and rare variants may contribute to the disease independently although it does not disregard a possible joint effect as stated above. Exhaustive functional analysis of these variants would be out of the scope of this study and although it could back up the bioinformatics prediction “*in vitro*” data alone will not ensure casualty. This study conveys the problematic assessment of the role of rare variants in disease and emphasises the difficulty in establishing genotype-phenotype correlations. Yet, providing a mutation profile for such a large number of patients will eventually help elucidate the architecture of the disease.

## Materials and Methods

The study was approved by the institutional review board of The University of Hong Kong together with the Hospital Authority (IRB:UW06-349/T/1374). Blood samples were drawn from all participants after obtaining informed consent (parental consent in newborns and children below age 7).

### Patients and controls

A total of **601** HSCR patients (Males = 490; Females = 111; M∶F = 4.4∶1) recruited throughout Hong Kong and Mainland China were included in the study. Four patients (C16C/C48C/HKC2C/HK75C) reported having a distant relative with the disease, but never parents. Diagnosis was based on histological examination of either biopsy or surgical resection material for absence of enteric plexuses. Sixty-seven patients were syndromic and among those, 16 had Down syndrome (Table 1).

Among the **601** HSCR patients, 430 patients had been genotyped for 21 *RET* SNPs across a ∼60 kb region of the *RET* gene as we previously described [4], 258 had been included in our previous genome-wide association study (GWAS) on Chinese HSCR (discovery phase/replication) [20] and 192 in the International Hirschsprung disease study on the differential contributions of *RET* mutations to Hirschsprung disease liability [2]. The *RET* rare variants for 86 of the 601 HSCR patients had been previously reported [12]. As controls, 250 ethnically matched individuals (Males = 160; Females = 90) with no diagnosis of HSCR were included.

### Sequencing of the *RET* coding regions

The *RET* 21 exons were screened in patients, parents when available, and controls to avoid the ascertainment bias caused when only the exons with variants identified in patients are sequenced in controls as previously described [12].

### Bioinformatics analysis

Seattle SEQ annotation version 5.06 and dbSNP-Q were used for annotation purposes and queries on dbSNP, HapMap and 1000 Genomes project databases. Polyphen and SIFT (Sorting Intolerant From Tolerant; scores <0.05 = deleterious are considered deleterious) were used to assess the effect of non-synonymous/missense changes on the protein. Human Splicing Finder Version 2.4.1 (default) was used to investigate whether the nucleotide changes disrupted/created exonic splicing enhancers (ESEs) or silencers (ESS), or branch or splice-sites [24]. Default thresholds were used. To assess whether rare silent or missense variants could affect the secondary structure/stability of the RNA we used RNAmute. Variants to be submitted to RNAmute were selected according to 1) their level of conservation at both DNA and protein level and ii) by consulting/accessing the UCSC Evofold UCSC track (41way multi-alignment, hg19) [25]. Conservation was inferred from i) genomic base wise conservation scores calculated using PhyloP from the PHAST package (28-species multiple alignment) and from ii) the coded amino acid conservation examined using ConSurf web server. Among “silent” variants, those variants in the most conserved sites were thought to have potential functional relevance [26], [27].

### Haplotype reconstruction

In order to phase rare variants present in individuals heterozygous for the *RET* risk haplotype (comprising the functional rs2435357 *RET* intron 1 SNP), we used the genotypes of 21 *RET* SNP which generated for 430 patients in our preceding studies. The *RET* rare variants identified were used as a SNP (one at the time) to re-construct haplotypes comprising the rare allele using the statistical software package PHASE version 2 as previously described [2], [4], [20]. A relation of the 21 SNPs can be found in the Cornes *et al.* manuscript [4].

## Supporting Information

Figure S1 Schematic representation of the impact of the variants Q327Q, L465L, L1077L, L651L, P841P, G954G F961L, Y1062C on RNA secondary structure (see main text and Material S1).
(PDF)

Table S1 A: distribution of the 61 rare *RET* variants across genders. B: characteristics of the HSCR patients with the R114H variant (N = 38).
(DOCX)

Table S2 Bioinformatics prediction of the rare *RET* variants identified.
(DOCX)

Table S3 Distribution of the 55 CDS rare variants in the RET protein domains (excludes intronic or 5′UT variants).
(DOCX)

Table S4 Rare *RET* variants identified in HSCR patients.
(DOCX)

Material S1 (DOCX)
