# Supplementary material for: RET Mutational Spectrum in Hirschsprung Disease: Evaluation of 601 Chinese Patients
Source: PLoS One. 2011 Dec 9;6(12):e28986. doi: 10.1371/journal.pone.0028986 (PMC3235168; doi:10.1371/journal.pone.0028986)
Supplement: Figure S1 — Schematic representation of the impact of the variants Q327Q, L465L, L1077L, L651L, P841P, G954G F961L, Y1062C on RNA secondary structure (see main text and Material S1). (PDF) [file pone.0028986.s001.pdf]

Generated by Foxit PDF Creator © Foxit Software
http://www.foxitsoftware.com For evaluation only.

981G>A Q327Q

WildUype GCUCCCCGGGGACACCUGGGCCCAGCAGACCUUCCGGGUGGAACACUGGCCCAAC

MuUaUion GCUCCCCGGGGACACCUGGGCCCAGCAAACCUUCCGGGUGGAACACUGGCCCAAC

Wildtype

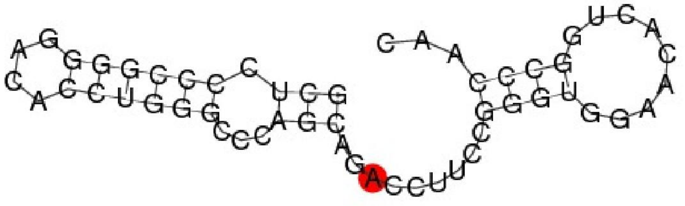

Mutation

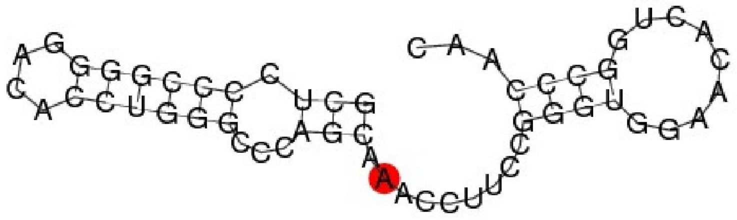

Wild-Uype free energy:-19.0 kcal/mol

MuUaUion free energy:-19.0 kcal/mol

Generated by Foxit PDF Creator © Foxit Software
http://www.foxitsoftware.com For evaluation only.

1393C>U L465L

UCAGCCGAGGACACCUCGGGGAUCCUGUUUGUGAAUGACACCAAGGCCCUGCGGC

UCAGCCGAGGACACCUCGGGGAUUCUGUUUGUGAAUGACACCAAGGCCCUGCGGC

Wildtype

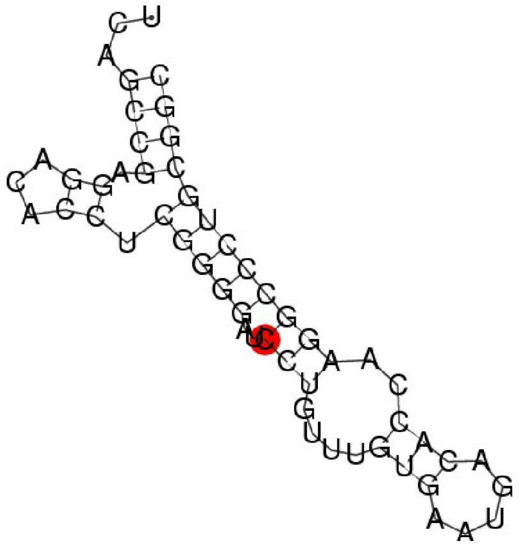

Mutation

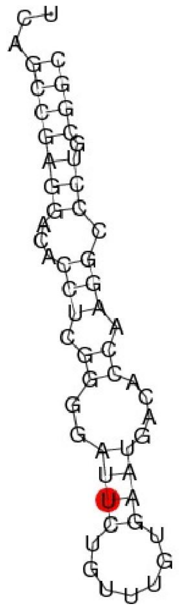

Wild-Uype free energy:-16.1 kcal/mol

MuUaUion free energy:-14.5 kcal/mol

Generated by Foxit PDF Creator © Foxit Software
http://www.foxitsoftware.com   For evaluation only.

3231 C>G   L1077L

CCGAACUGGCCUGGAGAGAGUCCUGUACCACUCACGAGAGCUGAUGGCACUAACACUGGGU

CCGAACUGGCCUGGAGAGAGUCCUGUACCACUGACGAGAGCUGAUGGCACUAACACUGGGU

Wildtype

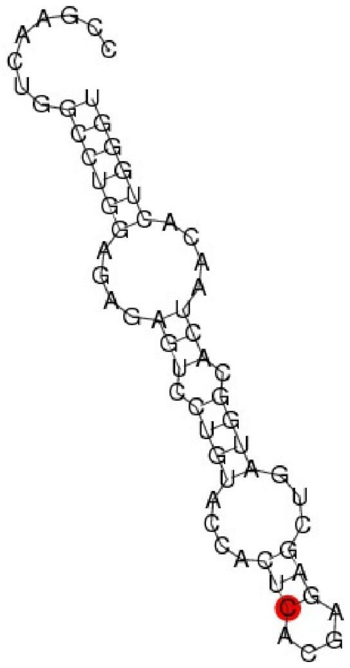

Mutation

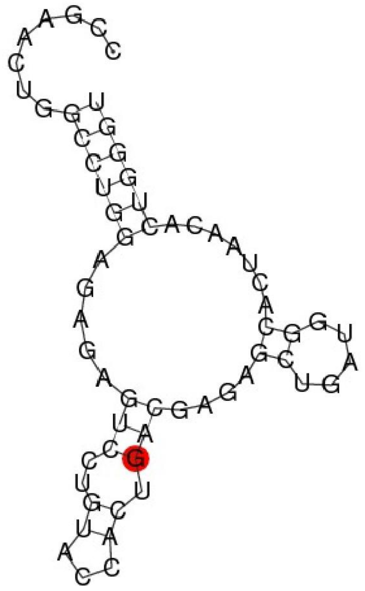

Wild-Uype free energy:-12.2 kcal/mol

MuUaUion free energy:-9.3 kcal/mol

Generated by Foxit PDF Creator © Foxit Software
http://www.foxitsoftware.com For evaluation only.

1953 G>A L651L

UCCUCUUCUCCUUCAUCGUCUCGGUGCUGCUGUCUGCCUUCUGCAUCCACUGCUACCACA

UCCUCUUCUCCUUCAUCGUCUCGGUGCUGCUAUCUGCCUUCUGCAUCCACUGCUACCACA

Wildtype

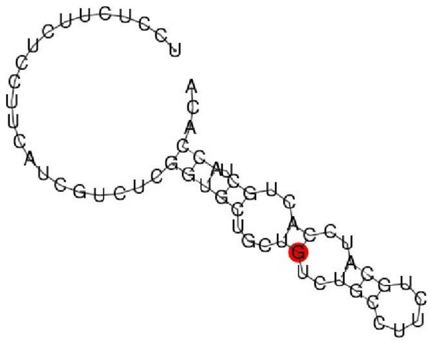

Mutation

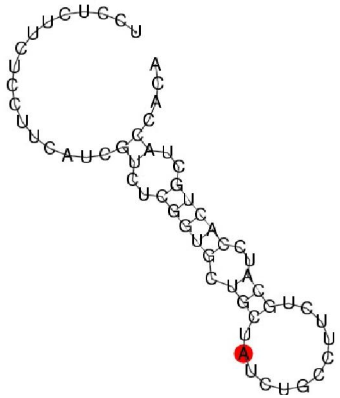

Wild-type free energy: -9.1 kcal/mol

Mutation free energy: -7.6 kcal/mol

Generated by Foxit PDF Creator © Foxit Software
http://www.foxitsoftware.com For evaluation only.

2523 G>A P841P

GCAGCCGCAACUCCAGCUCCCUGGACCACCCGGAUGAGCGGGCCCUCACCAUGGG

GCAGCCGCAACUCCAGCUCCCUGGACCACCCAGAUGAGCGGGCCCUCACCAUGGG

Wildtype

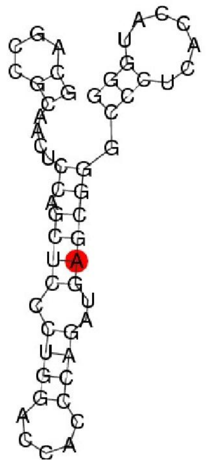

Mutation

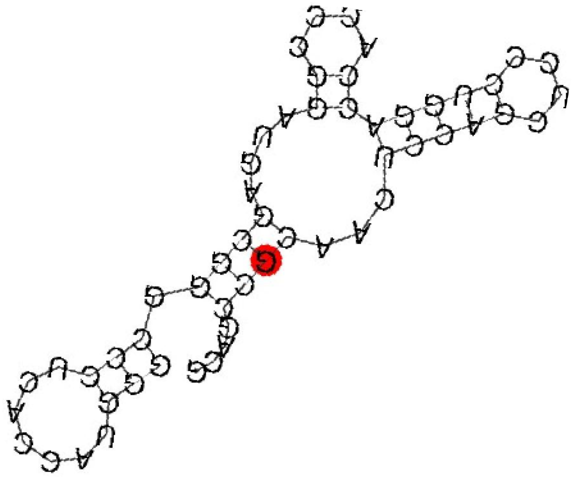

Wild-type free energy: -15.5 kcal/mo

Mutation free energy: -16.1kcal/mol

Generated by Foxit PDF Creator © Foxit Software
http://www.foxitsoftware.com For evaluation only.

2862 G>A G954G

GAUCGUGACCCUAGGGGGAAACCCCUAUCCUGGGAUUCCUCCUGAGCGGCUCUUCAACCU

GAUCGUGACCCUAGGGGGAAACCCCUAUCCUGGAAUUCCUCCUGAGCGGCUCUUCAACCU

Wildtype

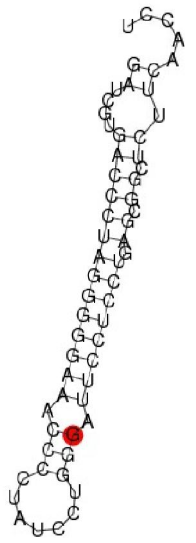

Mutation

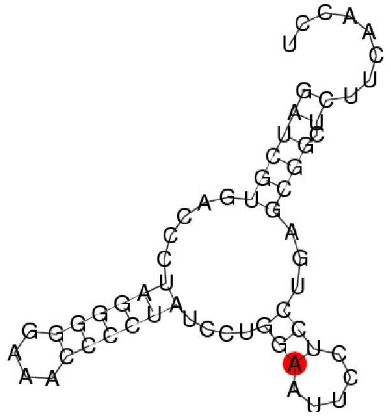

Wild-type free energy: -20.76 kcal/mol

Mutation free energy: -18.6 kcal/mol

Generated by Foxit PDF Creator © Foxit Software
http://www.foxitsoftware.com For evaluation only.

2881 T>C F961L

UCUUCAACCUUCUGAAGA

UCCUCAACCUUCUGAAGA

Evofold functional RNA substructure

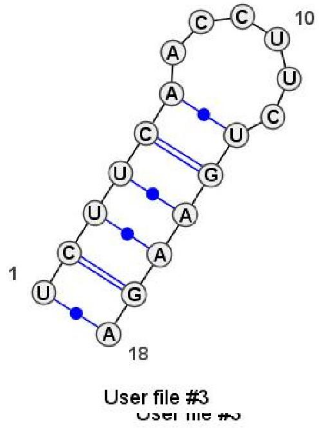

RNA substructure altered by mutation

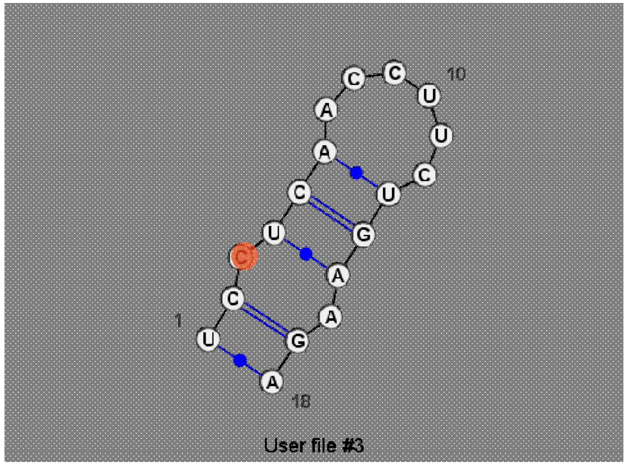

Wild-type free energy: -4.5 kcal/mol

Mutation free energy: -0.4 kcal/mol

Generated by Foxit PDF Creator © Foxit Software
http://www.foxitsoftware.com For evaluation only.

3185 A>G Y1062C

AGAAUCUAGUAAAUGCAUGGGAAAUUCUACCAUAGAGUUUGUUUU
AGAAUCUAGUAAGUGCAUGGGAAAUUCUACCAUAGAGUUUGUUUU

Evofold functional RNA substructure

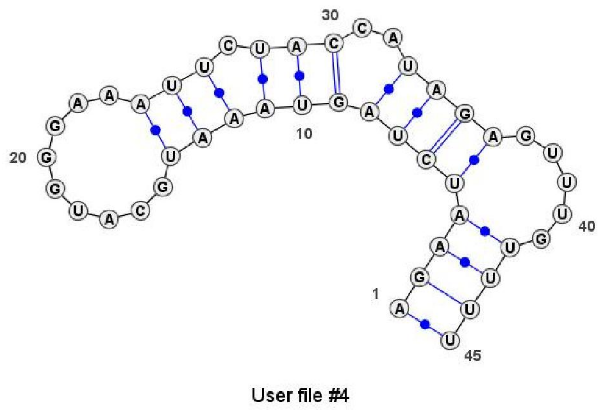

RNA substructure altered by mutation

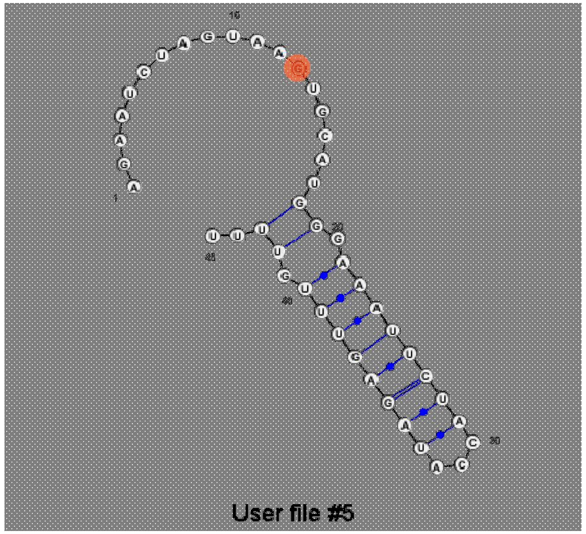

Wild-type free energy: -4.4 kcal/mol

Mutation free energy: -4.1kcal/mol
