# Supplementary material for: RET Mutational Spectrum in Hirschsprung Disease: Evaluation of 601 Chinese Patients
Source: PLoS One. 2011 Dec 9;6(12):e28986. doi: 10.1371/journal.pone.0028986 (PMC3235168; doi:10.1371/journal.pone.0028986)
Supplement: Table S1 — A: distribution of the 61 rare RET variants across genders. B: characteristics of the HSCR patients with the R114H variant (N = 38). (DOCX) [file pone.0028986.s002.docx]

|  | N | **%^a^** | **%^b^** |
| --- | --- | --- | --- |
| Total number of individuals mutated: | 100 [8] {1} | 16.64% | - |
| Mutated males: | 79 [5] {0} | 13.14% | 16.12% |
| Mutated females: | 21 [3] {1} | 3.49% | 18.92% |

Table S1a

N: number of individuals; **^a^**:% of the total number of patients; ^b^:% of the total number of patients of the same sex;

Table S1b

|  | S-HSCR | L-HSCR | Undetermined |
| --- | --- | --- | --- |
| Male | 23 | 2 | 6 |
| Female | 6 | 1 | - |
