# Supplementary material for: RET Mutational Spectrum in Hirschsprung Disease: Evaluation of 601 Chinese Patients
Source: PLoS One. 2011 Dec 9;6(12):e28986. doi: 10.1371/journal.pone.0028986 (PMC3235168; doi:10.1371/journal.pone.0028986)
Supplement: Table S2 — Bioinformatics prediction of the rare RET variants identified. (DOCX) [file pone.0028986.s003.docx]

| DNA | Protein | Sex | Phenotype | Conservation | | Functional prediction | | | | | |
| --- | --- | --- | --- | --- | --- | --- | --- | --- | --- | --- | --- |
| NM_020975.4 | NP_066124.1 |  |  | DNA | Protein | Polyphen | SIFT | Splice sites | ESE/ESS | Evofold | RNAmute |
| c.-37 G>C | NA | M | S | 0.179 | NA | NA | NA | NA | NA | No | NA |
| c.96 G>A | S32S | M | UD | 0.010 | 0.122 | NA | NA | No | ESE+;ESS+ | No | NA |
| **c.208C>T** | **Q70X** | **M** | **UD** | **0.677** | **1.579** | **NA** | **NA** | **NA** | **NA** | **No** | **NA** |
| c.337+5G>C | NA | M | L | 0.358 | NA | NA | NA | DS- | NA | No | NA |
| c.340C>T | R114C | M | UD | 0.360 | 2.915 | PRD | D | NA | NA | No | NA |
| c.341G>A | R114H^p,m^ | SM | SM | 0.678 | 2.915 | PSD | T | No | ESE- | No | NA |
| c.360C>T | T120T^D^ | F | S | 0.678 | 0.035 | NA | NA | No | ESE+;ESS- | No | NA |
| c.434T>G | V145G^D^ | F | TCA | 1.264 | -0.506 | PRD | D | NA | NA | No | NA |
| c.464C>T | P155L^m^ | M | S | 1.264 | -0.366 | PRD | D | NA | NA | No | NA |
| c.524G>C | R175P | M | S | 0.678 | -0.484 | PRD | T | No | ESE+;ESS+ | No | NA |
| **c.538C>T** | **R180X** | **F** | **L** | **0.181** | **0.072** | **NA** | **NA** | **NA** | **NA** | **No** | **NA** |
| c.687G>A | L229L^p^ | M | S | 0.360 | 0.245 | NA | NA | AS+ | ESE+/-;ESS+ | No | NA |
| **c.716_740del** | **L239PfsX8^P^** | **M** | **UD** | **1.264** | **-0.744** | **NA** | **NA** | **NA** | **NA** | **No** | **NA** |
| c.832A>C | T278P | M | L | 1.264 | -0.744 | PSD | T | No | ESE**-** | No | NA |
| c.832A>G | T278A | M | S/UD | 1.264 | -0.744 | PSD | T | No | ESE+/- | No | NA |
| c.833C>A | T278N | M | S | 0.678 | -0.744 | PSD | T | No | ESE-;ESS- | No | NA |
| c.874G>A | V292M | M | S/UD/S | 1.264 | -0.47 | PRD | D | NA | NA | No | NA |
| c.885G>A | T295T | M | TCA/UD | 0.002 | -0.524 | NA | NA | No | ESE+ | No | NA |
| c.898G>A | D300N | M | L | 1.264 | -0.744 | PRD | D | NA | NA | No | NA |
| **c.905_906insGCAG** | **N302EfsX53** | **F** | **TCA** | **0.678** | **-0.744** | **NA** | **NA** | **NA** | **NA** | **No** | **NA** |
| c.938G>A | R313Q | M | S | 0.360 | -0.436 | PSD | D | NA | NA | No | NA |
| c.947G>T | S316I | M | S | 0.032 | 1.034 | PSD | T | No | ESE- | No | NA |
| c.981G>A | Q327Q | M | S | 1.264 | 0.035 | NA | NA | AS- | ESE- | No | No |
| c.1016C>T | S339L | F | S | 0.181 | 1.088 | Benign | T | No | ESE- | No | NA |
| c.1057G>T | D353Y | M | S | 0.358 | 0.227 | PSD | D | NA | NA | No | NA |
| c.1079G>A | R360Q | M | UD | 0.360 | 0.134 | Benign | T | AS+ | ESE+/-;ESS+ | No | NA |
| c.1189G>A | V397M^f^ | M | L | 0.678 | -0.287 | PRD | T | No | ESS- | No | NA |
| c.1234G>A | V412M | M | S | 1.264 | -0.391 | PRD | T | AS+ | ESE- | No | NA |
| c.1267G>A | G423R | M | S | 0.678 | -0.383 | PRD | T | AS+ | ESE+;ESS- | No | NA |
| c.1353G>T | T451T | F | L | 0.181 | -0.056 | NA | NA | No | ESE+ | No | NA |
| **c.1385C>A** | **S462X^f^** | **M** | **S** | **0.678** | **1.463** | **NA** | **NA** | **NA** | **NA** | **No** | **NA** |
| c.1393C>T | L465L | M | UD | 1.264 | -0.308 | NA | NA | No | No | No | Yes |
| c.1438G>A | E480K | M | S | 0.360 | -0.227 | Benign | T | No | ESE- | No | NA |
| **c.1449delC** | **Y483X^D^** | **M** | **S** | **0.678** | **-0.495** | **NA** | **NA** | **NA** | **NA** | **No** | **NA** |
| **c.1549delC** | **L517CfsX121** | **M** | **UD** | **0.358** | **0.631** | **NA** | **NA** | **NA** | **NA** | **No** | **NA** |
| **c.1643_1648+4del** | **K549_G550del^D^** | **F** | **TCA** | **1.264** | **-0.101** | **NA** | **NA** | **DS-** | **NA** | **No** | **NA** |
| c.1760-2_-1delAG | NA | F | UD | 1.264 | NA | NA | NA | AS- | NA | No | NA |
| c.1783G>C | E595Q | M | S | 0.677 | 0.13 | PSD | T | AS+ | ESE+;ESS- | No | NA |
| **c.1908delG** | **V636fsX1^D^** | **M** | **TCA** | **0.032** | **-0.147** | **NA** | **NA** | **NA** | **NA** | **No** | **NA** |
| c.1920C>T | A640A^p^ | M | S | 0.002 | 0.345 | NA | NA | No | ESE-;ESS- | No | NA |
| c.1953G>A | L651L | M | S | 0.181 | -0.744 | NA | NA | No | ESS- | No | Yes |
| c.2001A>T | P667P | M | S | 0.012 | -0.433 | NA | NA | No | ESE+ | No | NA |
| c.2036C>T | P679L | M | UD | 0.678 | -0.529 | PRD | T | No | ESE-;ESS+ | No | NA |
| c.2081G>A | R694Q^p^ | M | S | 0.360 | -0.538 | PRD | T | AS+ | ESE-;ESS+ | No | NA |
| c.2136+15_+36del | NA | M | S | 0.360 | NA | NA | NA | BP- | NA | No | NA |
| c.2191_2193delGGA | G731del^m^ | F | TIA | 1.264 | -0.744 | NA | NA | AS- | ESE+;ESS+/- | No | NA |
| c.2285-30G>A | NA | M | TCA | 0.035 | NA | NA | NA | No | NA | No | NA |
| c.2348A>G | N783S^m^ | M | S | 1.264 | -0.496 | PRD | T | AS+ | No | No | NA |
| c.2392+58A>G | NA | M | UD | 0.174 | NA | NA | NA | BP- | NA | No | NA |
| c.2392+103C>T | NA | M | UD | 0.679 | NA | NA | NA | No | NA | No | NA |
| c.2488G>A | G830R | M | UD | 0.352 | -0.033 | PRD | T | AS+ | ESE-;ESS- | No | NA |
| c.2523G>A | P841P | F | UD | 0.005 | -0.744 | NA | NA | AS+;DS+ | ESE+/-;ESS+ | No | Yes |
| c.2547C>T | G849G | F | S | 0.074 | -0.454 | NA | NA | DS+ | No | No | NA |
| c.2720A>C | K907T | M | UD | 1.264 | -0.744 | PRD | D | NA | NA | No | NA |
| c.2862G>A | G954G^D^ | F | S | 0.179 | -0.744 | NA | NA | No | ESE-;ESS- | No | Yes |
| c.2881T>C | F961L^D^ | M | S | 1.264 | -0.452 | PRD | D | NA | NA | Yes | Yes |
| **c.3148C>T** | **R1050X** | **F** | **S** | **0.680** | **-0.145** | **NA** | **NA** | **NA** | **NA** | **No** | **NA** |
| c.3154C>G | L1052V | F | S | 0.680 | -0.744 | PRD | T | No | ESE+/- | No | NA |
| c.3185A>G | Y1062C^f^ | M | L | 0.680 | 0.142 | PRD | D | NA | NA | Yes | Yes |
| c.3191T>C | M1064T | M | S | 1.264 | -0.272 | PRD | T | No | ESE+ | No | NA |
| c.3231C>G | L1077L | F | UD | 1.264 | -0.744 | NA | NA | No | ESE+/-;ESS+/- | No | Yes |

m: maternal inheritance; p: paternal inheritance; D: *de novo*; f: familial involvement reported; M: male; F: female; UD: undetermined; NA: not applicable; No: no alteration detected; PRD: probably damaging; PSD: possibly damaging; D: damaging; T: tolerated; +:created; -:broken; ESE/ESS:exonic splicing enhancer/silencer; BP: branchpoint; AS: acceptor site; DS: donor site;
