# Supplementary material for: RET Mutational Spectrum in Hirschsprung Disease: Evaluation of 601 Chinese Patients
Source: PLoS One. 2011 Dec 9;6(12):e28986. doi: 10.1371/journal.pone.0028986 (PMC3235168; doi:10.1371/journal.pone.0028986)
Supplement: Table S3 — Distribution of the 55 CDS rare variants in the RET protein domains (excludes intronic or 5′UT variants). (DOCX) [file pone.0028986.s004.docx]

| RET domains | | Residues | Miss-sense (N=29) | | | Nonsense-Frame-shift (N=12) | | | Silent (N=14) | | |
| --- | --- | --- | --- | --- | --- | --- | --- | --- | --- | --- | --- |
|  |  |  | S-HSCR (N=18) | L/TCA^a^ (N=6) | NA (N=5) | S-HSCR (N=4) | L/TCA^b^ (N=4) | NA (N=4) | S-HSCR (N=8) | L/TCA (N=2) | NA (N=4) |
| Extracellular N= 36 | Cadherin N= 32 | 28-516 | 12 | 4 | 2 | 3 | 2 | 2 | 3 | 2 | 2 |
|  | Cystein N= 4 | 517-635 | 1 |  |  |  | 1 | 2 |  |  |  |
| Transmembrane N= 3 | | 636-657 |  |  |  |  | 1 |  | 2 |  |  |
| Intracellular N= 16 |  | 658-723 | 1 |  | 1 |  |  |  | 1 |  |  |
|  | Tyrosine kinase N= 8 | 724-1016 | 2 | 1 | 2 |  |  |  | 2 |  | 1 |
|  |  | 1016-1114 | 2 | 1 |  | 1 |  |  |  |  | 1 |

^a^:includes G731del in frame deletion; ^b^:includes K549_G550del (*de* *novo* 10 bp deletion that affects both exon and intron 8); the only variant identified in the 5’UT is not included. R114H, T278A, V292M and T295T were found in more than one patient and in more than one phenotype.
