# Supplementary material for: RET Mutational Spectrum in Hirschsprung Disease: Evaluation of 601 Chinese Patients
Source: PLoS One. 2011 Dec 9;6(12):e28986. doi: 10.1371/journal.pone.0028986 (PMC3235168; doi:10.1371/journal.pone.0028986)
Supplement: Table S4 — Rare RET variants identified in HSCR patients. (DOCX) [file pone.0028986.s005.docx]

| Location | DNA | dbSNP/1000G^#^ | Protein | Exon | Number of patients/rs2435357 genotypes/(ID) | Sex | Phenotype | RV on risk haplotype | References (SM) |
| --- | --- | --- | --- | --- | --- | --- | --- | --- | --- |
| Build 37.1 | NM_020975.4 | Build 131 | NP_066124.1 |  |  |  |  |  |  |
| 43595607 | c.-37 G>C | No | - | 5'UT | 1*# (HK97C)^S8^ | M | S | NP | Garcia-Barcelo (2004) |
| 43595929 | c.96 G>A | No | S32S | 2 | 1* (C432C) | M | UD | Yes |  |
| 43596041 | c.208 C>T | No | Q70X | 2 | 1* (C436C) | M | UD | Yes |  |
| 43596175 | c.337+5G>C | No | - | (2) | 1*# (C441C) | M | L | NP |  |
| 43597792 | c.340 C>T | No | R114C | 3 | 1* (C424C) | M | UD | Yes |  |
| 43597793 | c.341 G>A | rs76397662 | R114H^p, m^ | 3 | 38 (34*/2*#)^S1,S2,S3^ | SM | SM | Yes (all) | Kanai (2002) Garcia-Barcelo (2004) |
| 43597812 | c.360 C>T | No | T120T^D^ | 3 | 1# (HK55C) | F | S | No | Garcia-Barcelo (2004) |
| 43597886 | c.434 T>G | No | V145G^D^ | 3 | 1* (HK66C) | F | TCA | Yes | Garcia-Barcelo (2004) |
| 43597916 | c.464 C>T | No | P155L^m^ | 3 | 1*# (HD13C) | M | S | No |  |
| 43597976 | c.524 G>C | No | R175P | 3 | 1# (C227C) | M | S | No |  |
| 43597990 | c.538 C>T | rs76449634 | R180X | 3 | 1* (HK137C) | F | L | Yes | Edery (1994) |
| 43600461 | c.687 G>A | No | L229L^p^ | 4 | 1* (HD07C) | M | S | Yes |  |
| 43600490 | c.716_740del | No | L239PfsX8^P^ | 4 | 1# (C498C) | M | UD | No |  |
| 43600606 | c.832 A>C | No | T278P | 4 | 1* (C284C) | M | L | Yes |  |
| 43600606 | c.832 A>G | No | T278A | 4 | 3* (HK100C, C493C, C509C) | M | S/UD/UD | Yes |  |
| 43600607 | c.833 C>A | rs35118262/Y | T278N | 4 | 2 (#223C,*#C519C) | M | S/S | No/NP | Sangkhathat (2006) |
| 43601830 | c.874 G>A | rs34682185 | V292M | 5 | 3*# (HK92, HK130, C254C) | M | S/UD/S | Yes/All | Castellone (2010) |
| 43601841 | c.885 G>A | No | T295T | 5 | 2*# (C23C,C358C) | M | TCA/UD | No |  |
| 43601854 | c.898 G>A | No | D300N | 5 | 1* (C260C) | M | L | Yes |  |
| 43601861 | c.905_906insGCAG | No | N302EfsX53 | 5 | 1*# (C371C) | F | TCA | NP |  |
| 43601894 | c.938 G>A | rs77702891 | R313Q | 5 | 1*# (C216C) | M | S | No | Seri (1997) |
| 43601903 | c.947 G>T | No | S316I | 5 | 1*# (C251C) | M | S | No |  |
| 43601937 | c.981 G>A | No | Q327Q | 5 | 1* (C17C) | M | S | Yes |  |
| 43601972 | c.1016 C>T | No | S339L | 5 | 1* (C348C) | F | S | Yes |  |
| 43602013 | c.1057 G>T | No | D353Y | 5 | 1* (C261C) | M | S | Yes |  |
| 43604494 | c.1079 G>A | No | R360Q | 6 | 1*# (C528C) | M | UD | NP |  |
| 43604604 | c.1189 G>A | No | V397M^f^ | 6 | 1*# (C16C)^+^ | M | L | No |  |
| 43604649 | c.1234 G>A | No | V412M | 6 | 1* (C47C) | M | S | Yes |  |
| 43606658 | c.1267 G>A | No | G423R | 7 | 1* (C180C) | M | S | Yes |  |
| 43606744 | c.1353 G>T | No | T451T | 7 | 1* (C242C) | F | L | Yes |  |
| 43606776 | c.1385 C>A | No | S462X^f^ | 7 | 1*# (C48C) | M | S | No |  |
| 43606784 | c.1393C>T | No | L465L | 7 | 1* (C503C)**^+^** | M | UD | Yes |  |
| 43606829 | c.1438 G>A | No | E480K | 7 | 1* (C4C) | M | S | Yes | Julies (2001) |
| 43606840 | c.1449delC | No | Y483X^D^ | 7 | 1* (HK26C) | M | S | Yes | Garcia-Barcelo (2004) |
| 43607573 | c.1549delC | No | L517CfsX121 | 8 | 1# (C504C) | M | UD | No |  |
| 43607667 | c.1643_1648+4del | No | K549_G550del^D^ | 8 | 1*# (HK83C) | F | TCA | NP | Garcia-Barcelo (2004) |
| 43609002 | c.1760-2_-1delAG | No | - | (9) | 1# (C500C) | F | UD | No |  |
| 43609027 | c.1783 G>C | No | E595Q | 10 | 1*# (C46C) | M | S | No |  |
| 43609956 | c.1908delG | No | V636fsX1^D^ | 11 | 1*# (HKC1C) | M | TCA | NP | Garcia-Barcelo (2004) |
| 43609968 | c.1920 C>T | No | A640A^p^ | 11 | 1* (HK106C) | M | S | Yes |  |
| 43610001 | c.1953 G>A | No | L651L | 11 | 1* (C24C) | M | S | Yes |  |
| 43610049 | c.2001 A>T | No | P667P | 11 | 1* (C6C) | M | S | Yes |  |
| 43610084 | c.2036 C>T | No | P679L | 11 | 1* (C299C) | M | UD | Yes |  |
| 43610129 | c.2081 G>A | No | R694Q^p^ | 11 | 1* (HK32C) | M | S | Yes | Garcia-Barcelo (2004) |
| 43610199 | c.2136+15_+36del | No | - | (11) | 1* (HK1C)^S4^ | M | S | Yes | Garcia-Barcelo (2004) |
| 43612086 | c.2191_2193delGGA | No | G731del^m^ | 12 | 1# (HK149C)^S5^ | F | TIA | No |  |
| 43613791 | c.2285-30G>A | No | - | (12) | 1* (C219C) | M | TCA | Yes |  |
| 43613884 | c.2348 A>G | No | N783S^m^ | 13 | 1*# (HK122C) | M | S | Yes |  |
| 43613986 | c.2392+58A>G | No | - | (13) | 1* (C501C) | M | UD | Yes |  |
| 43614031 | c.2392+103C>T | No | - | (13) | 1* (C503C)^+^ | M | UD | Yes |  |
| 43615074 | c.2488 G>A | No | G830R | 14 | 1*# (C293C) | M | UD | No |  |
| 43615109 | c.2523 G>A | rs56195026 | P841P | 14 | 1*# (C363C) | F | UD | NP |  |
| 43615133 | c.2547 C>T | No | G849G | 14 | 1# (C69C)^+^ | F | S | No |  |
| 43615641 | c.2720A>C | No | K907T | 15 | 1* (C516C) | M | UD | Yes |  |
| 43619179 | c.2862 G>A | No | G954G^D^ | 17 | 1# (HK55C) | F | S | No | Garcia-Barcelo (2004) |
| 43619198 | c.2881 T>C | No | F961L^D^ | 17 | 1# (HK25C)^S6^ | M | S | Yes |  |
| 43622131 | c.3148 C>T | No | R1050X | 19 | 1# (C69C)^+^ | F | S | No |  |
| 43622137 | c.3154 C>G | No | L1052V | 19 | 1* (C287C) | F | S | Yes |  |
| 43622168 | c.3185 A>G | No | Y1062C^f^ | 19 | 1*# (C16C)^+^ | M | L | No | Wu (2005); Ruiz (2006) |
| 43623563 | c.3191 T>C | No | M1064T | 20 | 1* (HK17C)^S7^ | M | S | Yes | Attie (1995) |
| 43623603 | c.3231 C>G | No | L1077L | 20 | 1*# (C492C) | F | UD | NP |  |

^#^ :1000G release March 2011; m: maternal inheritance; p: paternal inheritance; D: *de novo*; f: familial involvement reported; (): intron; *:TT; #:CC; *#:CT; S1-S8: syndromic patients UD: undetermined; M: male; F: female; + individual with 2 rare variants; NP: rare variant not phased with the *RET* risk haplotype; S1: rudimentary polydactyly; S2: Down and severe bilateral hearing loss; S3: sensorineural hearing loss; S4: mental retardation; S5: ectopic left kidney; S6: Vesicular-uretral reflux; respiratory problems; S7: soft eye ball; S8: Ondine’s association (negative for *PHOX2B* mutations); SM: supplementary material.
