# Supplementary material for: RET Mutational Spectrum in Hirschsprung Disease: Evaluation of 601 Chinese Patients
Source: PLoS One. 2011 Dec 9;6(12):e28986. doi: 10.1371/journal.pone.0028986 (PMC3235168; doi:10.1371/journal.pone.0028986)
Supplement: Material S1 — (DOCX) [file pone.0028986.s006.docx]

***Supplementary methods on bioinformatics analysis***

**i. Conservation Scores** were calculated by PhyloP from PHAST package [1] and ConSurf web server [[2](#_ENREF_2)]. Both calculations were based on the phylogenetic tree, which is superior to the algorithm using direct multiple sequence alignment (MSA), but still more or less would be affected by the property of sequence alignment of the region in the prescribed conservation score.

PhyloP applied the maximum likelihood method and the scores are obtained from a per-base calculation of the –log (p-value) from multiple species sequence alignment under a null hypothesis of neutral evolution. The score ranges from 0 to infinity, although in practice there is a maximum achievable value for any particular data set, which reached 1.264 in Placental Mammal base-wise annotation within the genomic range of *RET*.

The *RET* amino acid conservation score was calculated by ConSurf web server using empirical Bayesian method. The server allows for a maximum of 50 homologue sequences for alignment. The conservation score calculated would correspond to the evolutionary rate of each site, which means the lower evolutionary rate would be more conserved, and *vice versa.* The scores were represented after normalization, hence, a minus value would represent conservation, and the lowest score represents the most conserved position in a protein.

**ii.** **Pathogenicity of amino acid substitution** were predicted by SIFT (sorting intolerant from tolerant) [[3](#_ENREF_3),[4](#_ENREF_4)] and PolyPhen2 (Polymorphism Phenotyping v2) [[4](#_ENREF_4)].

SIFT uses sequence homology to predict whether an amino acid substitution will affect protein function. It constructs an MSA and considers the position of the missense variant and the type of the amino acid change. Based on the amino acids appearing at each position in the MSA, SIFT calculates the probability that a missense variant is tolerated conditional on the most frequent amino acid being tolerated. The amino acid substitution is predicted damaging if the propability is less than or equal to 0.05, and tolerated if the score is > 0.05. By this criterion, SIFT was tested to have a sensitivity of 68% and specificity of 62% [[5](#_ENREF_5)].

Polyohen2 utilizes a combination of sequence- and structure-based attributes for the description of an amino acid substitution, and the effect of mutation is predicted by a naive Bayesian classifier. PolyPhen-2 extracts the sequence and structure-based features of the substitution site and feeds them to a probabilistic classifier. It uses 5% - 10% false discovery rate (FPR) for HumDiv model as the thresholds for this ternary classification: probably damaging, possibly damaging and benign. Sensitivity of the prediction based on this criterion is 86%, and specificity 51% based on Janita Thusberg and collegues’ study [5].

**iii.** **Effect of the mutation on RNA substructure** was firstly predicted basing on the free energy minimization (RNA substructure) prediction method (RNAMute), by which the scale of the alteration induced by the mutations was evaluated by the distance of mutated RNA secondary structure to the wild-type structure [6]. Also, we evaluated the functionality of the mutations in RNA substructures formation by direct intersecting with the Evofold predicted RNA secondary structures [7].

The core utility of RNAMute is based on Vienna RNA package, which performs the RNA secondary structure predictions based on the minimum free energies theory of RNA structure formation. We applied the free energy change and Shapiro’s distance to evaluate the alteration introduced by mutation to the wild type RNA secondary structure [6].

Evofold is a comparative method for identifying functional RNA structures in multiple-sequence alignments. It is based on a probabilistic model-construction called a phylo-SCFG and exploits the characteristic differences of the substitution process in stem-pairing and unpaired regions to make its predictions. For the 48479 functional structure predicted, the authors estimate the precision in this prediction set is around 38%.

**Supplementary Results**

***Rare variants reported in other HSCR series***

Unfortunately, information of how those previously reported mutations arose (inherited or *de novo*) or the full characteristics of the patients bearing them was not always available. **R180X** was reported in two related Caucasian cases [8]. **T278N** was reported in a Thai S-HSCR patient [9] (not stated whether the variant was inherited or *de novo*). **R313Q** was found in homozygosity in a Caucasian TCA child with small bowel involvement), born of consanguineous parents [10]. **E480K** was also identified in two unrelated coloured S-HSCR individuals from South Africa (not stated whether the variant was inherited or *de novo)* [11]. **Y1062C** was reported in identical Taiwanese male twins of Chinese ancestry with S-HSCR [12] and in a Spanish/Caucasian S-HSCR who had inherited from his/her unaffected father,[13]; the gender of the probands reported with this mutation was not available. **M1064T** was identified in a S-HSCR familial case [14].

V292M has recently been reported as a germ-line *de novo* event in a patient with phaeochromocytoma and medullary thyroid carcinoma, a condition also due to DNA alterations in *RET* [15]. *In vitro* functional characterization indicated that V292M is a gain-of-function “mutation” with some degree of oncogenic potential. The bioinformatics prediction for V292M is damaging.

R114H was described in 2002 in a Japanese patient with CCHS, condition that has an incidence of 1.9% among HSCR patients. None of the HSCR patients with R114H presented CCHS features [16]. The only HSCR patient with CCHS (HK97C) had a 5'UT c.-37 G>C change as we previously reported [17]. This patient was negative for causal variants in *PHOX2B*, the main gene implicated in CCSH [18].

Some mutations previously reported by others are also found in more than one patient in our series. These are R114H (found patients with either S or L HSCR aganglionosis –supplementary Table 3), T278N identified in two patients with S-HSCR and, V292M identified in 3 patients (2 with S-HSCR and one with aganglionosis length undetermined). Recurrent mutations found only in the present study and never reported before are T278A, found in 3 patients (2 with S-HSCR and one with aganglionosis length undetermined) and T295T, found in a patient with TCA and in one with aganglionosis length undetermined.

***RET rare variants are not necessarily on the RET HSCR-risk haplotype***

Of these **23** heterozygous patients, we were able to phase their mutation with the haplotype for **15,** as **8** individuals had not been included in our previous *RET* genotyping exercise. Among those 15 phased heterozygous patients, 7 had the CDS-RVs on the risk haplotype(T) and 8 on the wild-type(C). Consequently, among the 92 patients with CDS-RVs phased (68TT+9CC+15CT=92), 17 (18.48%; 9CC+8C-phased) had the CDS-RVs on the non-risk-haplotype and 75 (81.52%; 68TT+7T-phased) on the risk-haplotype.

1. Murphy WJ, Eizirik E, O'Brien SJ, Madsen O, Scally M, et al. (2001) Resolution of the early placental mammal radiation using Bayesian phylogenetics. Science 294: 2348-2351.

2. Ashkenazy H, Erez E, Martz E, Pupko T, Ben-Tal N (2010) ConSurf 2010: calculating evolutionary conservation in sequence and structure of proteins and nucleic acids. Nucleic Acids Res 38: W529-533.

3. Ng PC, Henikoff S (2003) SIFT: Predicting amino acid changes that affect protein function. Nucleic Acids Res 31: 3812-3814.

4. Adzhubei IA, Schmidt S, Peshkin L, Ramensky VE, Gerasimova A, et al. (2010) A method and server for predicting damaging missense mutations. Nat Methods 7: 248-249.

5. Thusberg J, Olatubosun A, Vihinen M (2011) Performance of mutation pathogenicity prediction methods on missense variants. Hum Mutat 32: 358-368.

6. Churkin A, Barash D (2008) An efficient method for the prediction of deleterious multiple-point mutations in the secondary structure of RNAs using suboptimal folding solutions. BMC Bioinformatics 9: 222.

7. Pedersen JS, Bejerano G, Siepel A, Rosenbloom K, Lindblad-Toh K, et al. (2006) Identification and classification of conserved RNA secondary structures in the human genome. PLoS Comput Biol 2: e33.

8. Edery P, Lyonnet S, Mulligan LM, Pelet A, Dow E, et al. (1994) Mutations of the RET proto-oncogene in Hirschsprung's disease. Nature 367: 378-380.

9. Sangkhathat S, Kusafuka T, Chengkriwate P, Patrapinyokul S, Sangthong B, et al. (2006) Mutations and polymorphisms of Hirschsprung disease candidate genes in Thai patients. Journal of human genetics 51: 1126-1132.

10. Seri M, Yin L, Barone V, Bolino A, Celli I, et al. (1997) Frequency of RET mutations in long- and short-segment Hirschsprung disease. HumMutat 9: 243-249.

11. Julies MG, Moore SW, Kotze MJ, du PL (2001) Novel RET mutations in Hirschsprung's disease patients from the diverse South African population. EurJHumGenet 9: 419-423.

12. Wu TT, Tsai TW, Chu CT, Lee ZF, Hung CM, et al. (2005) Low RET mutation frequency and polymorphism analysis of the RET and EDNRB genes in patients with Hirschsprung disease in Taiwan. J Hum Genet 50: 168-174.

13. Ruiz-Ferrer M, Fernandez RM, Antinolo G, Lopez-Alonso M, Eng C, et al. (2006) A complex additive model of inheritance for Hirschsprung disease is supported by both RET mutations and predisposing RET haplotypes. Genet Med 8: 704-710.

14. Attie T, Pelet A, Edery P, Eng C, Mulligan LM, et al. (1995) Diversity of RET proto-oncogene mutations in familial and sporadic Hirschsprung disease. HumMolGenet 4: 1381-1386.

15. Castellone MD, Verrienti A, Magendra Rao D, Sponziello M, Fabbro D, et al. (2010) A novel de novo germ-line V292M mutation in the extracellular region of RET in a patient with phaeochromocytoma and medullary thyroid carcinoma: functional characterization. Clin Endocrinol (Oxf) 73: 529-534.

16. Kanai M, Numakura C, Sasaki A, Shirahata E, Akaba K, et al. (2002) Congenital central hypoventilation syndrome: a novel mutation of the RET gene in an isolated case. Tohoku JExpMed 196: 241-246.

17. Garcia-Barcelo M, Sham MH, Lee WS, Lui VC, Chen BL, et al. (2004) Highly Recurrent RET Mutations and Novel Mutations in Genes of the Receptor Tyrosine Kinase and Endothelin Receptor B Pathways in Chinese Patients with Sporadic Hirschsprung Disease. ClinChem 50: 93-100.

18. Amiel J, Laudier B, ttie-Bitach T, Trang H, de PL, et al. (2003) Polyalanine expansion and frameshift mutations of the paired-like homeobox gene PHOX2B in congenital central hypoventilation syndrome. NatGenet 33: 459-461.
